# Excitotoxicity increases the release of 24S-hydroxycholesterol via CYP46A1 activation

**DOI:** 10.1186/1750-1326-8-S1-O32

**Published:** 2013-09-13

**Authors:** Alejandro Sodero, Joris Vriens, Debapriya Ghosh, David Stegner, Anna Brachet, Marta Pallotto, Marco Sassoe-Pognetto, Jos Brouwers, Bernd Helms, Bernard Nieswandt, Thomas Voets, Carlos Dotti

**Affiliations:** 1 VIB Center for Biology of Disease, Katholieke Universiteit Leuven, Leuven, Belgium; 2 Department of Molecular Cell Biology, Katholieke Universiteit Leuven, Leuven, Belgium; 3 Rudolf Virchow Center for Experimental Biomedicine, University of Würzburg, Würzburg, Germany; 4 Centro de Biología Molecular Severo Ochoa, CSIC-UAM, Madrid, Spain; 5 Department of Anatomy, Pharmacology and Forensic Medicine, University of Turin and National Institute of Neuroscience, Torino, Italy; 6 Department of Biochemisty and Cell Biology, Faculty of Veterinary Medicine, University of Utrecht, Utrecht, The Netherlands; 7 Currently at Institute of Neuroscience, Université Catholique de Louvain, Brussels, Belgium

## Background

Excitotoxicity, a common hallmark of different neurological disorders including Alzheimer’s disease (AD), is the consequence of exacerbated neuronal stimulation and leads to a high influx of calcium trough membrane glutamate receptors [1].

On the other hand, high levels of the cholesterol metabolite 24S-hydroxycholesterol (24-HC) have been found in cerebral spinal fluid (CSF) from AD patients [2,3]. Brain cholesterol homeostasis is an essential, tightly-regulated process that ensures neuronal integrity, viability, and function. One of the mechanisms that neurons put to work to regulate the amount of cellular cholesterol is its conversion into the metabolite 24-HC by the 24-cholesterol hydroxylase CYP46A1 [4]. In this work, we explored the possibility that excitotoxicity, a common process that precedes neurodegeneration, could be a direct modulator of the 24-HC production via a CYP46A1-mediated hydroxylation of cholesterol.

## Materials and methods

Cholesterol levels were measured by fluorimetric detection (Amplex Red Kit, Invitrogen) in the 100,000g membrane fraction of cultured hippocampal neurons and mouse hippocampal tissue, and in purified synaptosomes.

- 24-HC release was measured in the medium of cultured hippocampal neurons by LC/MS analysis.

- Cell surface biotinylation was performed using a membrane-impermeable biotin (EZ-link Sulfo-NHS-Biotin, Pierce), and cell surface CYP46A1 was detected by western immunoblotting using two different antibodies.

- STIM2 knockout mice were generated in the lab of Prof. Bernard. Nieswandt [5],

- Electron microscopy was performed in mouse hippocampal slices.

- TIRF microscopy was performed in HEK293T cells and hippocampal neurons expressing CYP46A1 fused to GFP.

- Intracellular calcium concentration was monitored in cultured hippocampal neurons using ratiometric Fura-2-based fluorimetry.

## Results

We observed that excessive stimulation of glutamate receptors induces a significant loss of membrane cholesterol, which is paralleled by the release to the extracellular milieu of the metabolite 24-HC. Cholesterol loss was induced by depolarization of cultured hippocampal neurons with high potassium or stimulation of postsynaptic NMDA receptors. Importantly, purified synapses showed a similar reduction of this sterol after *in vitro* stimulation. Moreover, we observed a significant reduction in the content of cholesterol of hippocampal membranes of C57BL/6J mice treated with kainic acid for only 30 minutes (supra-epileptic condition). Consistent with a cause-effect relationship, knockdown of CYP46A1 prevented the glutamate-mediated cholesterol loss in cultured hippocampal neurons. Mechanistically, we found that the cholesterol reduction requires high levels of intracellular calcium, a functional Stromal Interaction Molecule 2 (STIM2) and mobilization of CYP46A1 from the endoplasmic reticulum, its natural sub-cellular compartment, towards the plasma membrane. Imaging studies with Fura-2 showed that the cholesterol loss is able to modulate the intracellular calcium response induced by depolarization.

## Conclusions

This study underscores the key role of excitatory neurotransmission in the control of neuronal cholesterol content and suggests that excitotoxicity is one of the causes for the increased levels of 24-HC observed in the CSF of AD patients. Whether or not the observed cholesterol catabolism and the reduction in the magnitude of the calcium peaks that parallel excitotoxicity are part of a protective response to fight against injury remains elusive and merits further investigation.
